# Association between modified a body shape index and incident cognitive impairment in middle-aged and older adults: a prospective cohort study

**DOI:** 10.1186/s12889-026-28763-6

**Published:** 2026-08-17

**Authors:** Jingshuo Zhang, Qingqing Jiang, Furong Wang, Chen Yu

**Affiliations:** 1 School of Nursing, Tongji Medical College, Huazhong University of Science and Technology, Wuhan, Hubei Province 430000 China; 2 Department of Neurology, Tongji Hospital, Tongji Medical College, Huazhong University of Science and Technology, Wuhan, Hubei Province 430000 China; 3 School of Computer Science and Technology, Huazhong University of Science and Technology, Wuhan, Hubei Province 430074 China

**Keywords:** Modified A Body Shape Index, Cognitive impairment, Middle-aged and older adults, CHARLS, Prospective cohort study

## Abstract

**Background:**

Central obesity contributes significantly to cognitive decline. Traditional indices like BMI inadequately capture visceral adiposity and exhibit the obesity paradox in elderly populations.This study looked into the connection between a modified A Body Shape Index (ABSI) and the development of cognitive impairment in middle-aged and older adults.

**Methods:**

Longitudinal data from 4,353 eligible participants in the 2011–2020 CHARLS cohort were analyzed. Modified ABSI was calculated based on waist circumference, BMI, and height and categorized into quartiles. Cox proportional hazards regression models were used to evaluate the association between modified ABSI and incident cognitive impairment. The fully adjusted model included age, sex, residence, education, marital status, smoking, drinking, hypertension, diabetes mellitus, dyslipidemia, stroke, heart disease, systolic and diastolic blood pressure, triglycerides, LDL-C, HDL-C, HbA1c, CRP, serum creatinine, and uric acid. Restricted cubic spline, subgroup, and sensitivity analyses were also performed.

**Results:**

Over the follow-up period, cognitive impairment occurred in 1,822 participants, corresponding to 41.9% of the study population. In the Cox model that was fully adjusted, individuals in the highest ABSI quartile (Q4) exhibited a markedly elevated hazard of cognitive impairment relative to those in the lowest quartile (Q1) (HR = 1.22, *P* = 0.024). A pronounced dose–response pattern was evident, which persisted as statistically significant and linear following comprehensive covariate adjustment (*P* = 0.037). Subgroup analyses indicated more pronounced associations among female participants and individuals aged 70 years or older (*P* < 0.05).

**Conclusion:**

High ABSI levels are significantly associated with an increased long-term risk of cognitive impairment in middle-aged and older adults. The findings suggest that ABSI could serve as an effective surrogate marker for central adiposity, facilitating the early recognition of individuals at heightened risk for cognitive decline.

**Supplementary Information:**

The online version contains supplementary material available at https://doi.org/10.1186/s12889-026-28763-6.

## Introduction

Cognitive impairment has arisen as a major public health challenge within rapidly aging populations worldwide. It is predicted that the global dementia population will exceed 150 million by 2050, creating substantial socioeconomic challenges for healthcare infrastructures [1, 2]. In China, epidemiological investigations have estimated that approximately 39 million adults aged 60 years and above exhibit mild cognitive impairment, with around 15 million diagnosed with dementia [3, 4]. Given that neuropathological alterations frequently commence decades prior to the manifestation of overt clinical symptoms, the early detection of modifiable risk factors during the prodromal phase holds substantial importance for formulating efficacious preventive interventions [5, 6].

Central or visceral adiposity is progressively acknowledged as a pivotal contributor to neurodegenerative processes, primarily through pathways involving persistent low-grade systemic inflammation and insulin resistance [7, 8]. Although body mass index (BMI) has long served as the conventional indicator of obesity, its failure to distinguish adipose tissue from lean muscle mass has contributed to the debated “obesity paradox,” whereby elevated BMI appears paradoxically protective among older adults [9, 10]. To overcome these constraints, ABSI was introduced to offer a more accurate assessment of visceral fat distribution by normalizing waist circumference according to height and weight. Emerging evidence from both American and Chinese cohorts has linked higher ABSI values to diminished cognitive function [11, 12]. However, previous studies have predominantly used cross-sectional designs, which cannot establish whether elevated ABSI precedes subsequent cognitive impairment. This limitation is particularly important in middle-aged and older populations, in whom age-related weight loss, sarcopenia, frailty, and preclinical cognitive decline may complicate cross-sectional associations between adiposity and cognition. Moreover, although a modified ABSI has been developed to better characterize body shape in Chinese populations, evidence regarding its association with the long-term risk of incident cognitive impairment in initially cognitively unimpaired adults remains limited. Therefore, prospective evaluation is needed to determine whether baseline modified ABSI can identify individuals at increased risk of developing cognitive impairment over time.

To address these knowledge gaps, we used longitudinal data from the China Health and Retirement Longitudinal Study (CHARLS) to investigate the association between baseline modified ABSI and incident cognitive impairment over a 9-year follow-up period. By excluding participants with cognitive impairment at baseline and identifying newly occurring cognitive impairment during follow-up, the present study extends previous cross-sectional evidence by establishing a clear temporal sequence between modified ABSI exposure and subsequent cognitive outcomes. We further examined the dose–response relationship, potential effect modification across clinically relevant subgroups, and the robustness of the association through sensitivity analyses. Therefore, this study aimed to clarify the prospective relevance of modified ABSI as a simple anthropometric marker for identifying middle-aged and older adults at elevated risk of future cognitive impairment.

## Methods

### Study design and participants

This prospective cohort investigation drew upon longitudinal data from the China Health and Retirement Longitudinal Study (CHARLS), spanning the baseline assessment conducted in 2011 through the 2020 follow-up wave. CHARLS represents a biennial, nationally representative survey focusing on Chinese adults aged 45 years and older, implemented via a multistage, stratified, probability-proportional-to-size sampling strategy across 150 administrative divisions nationwide.

**Fig. 1:**
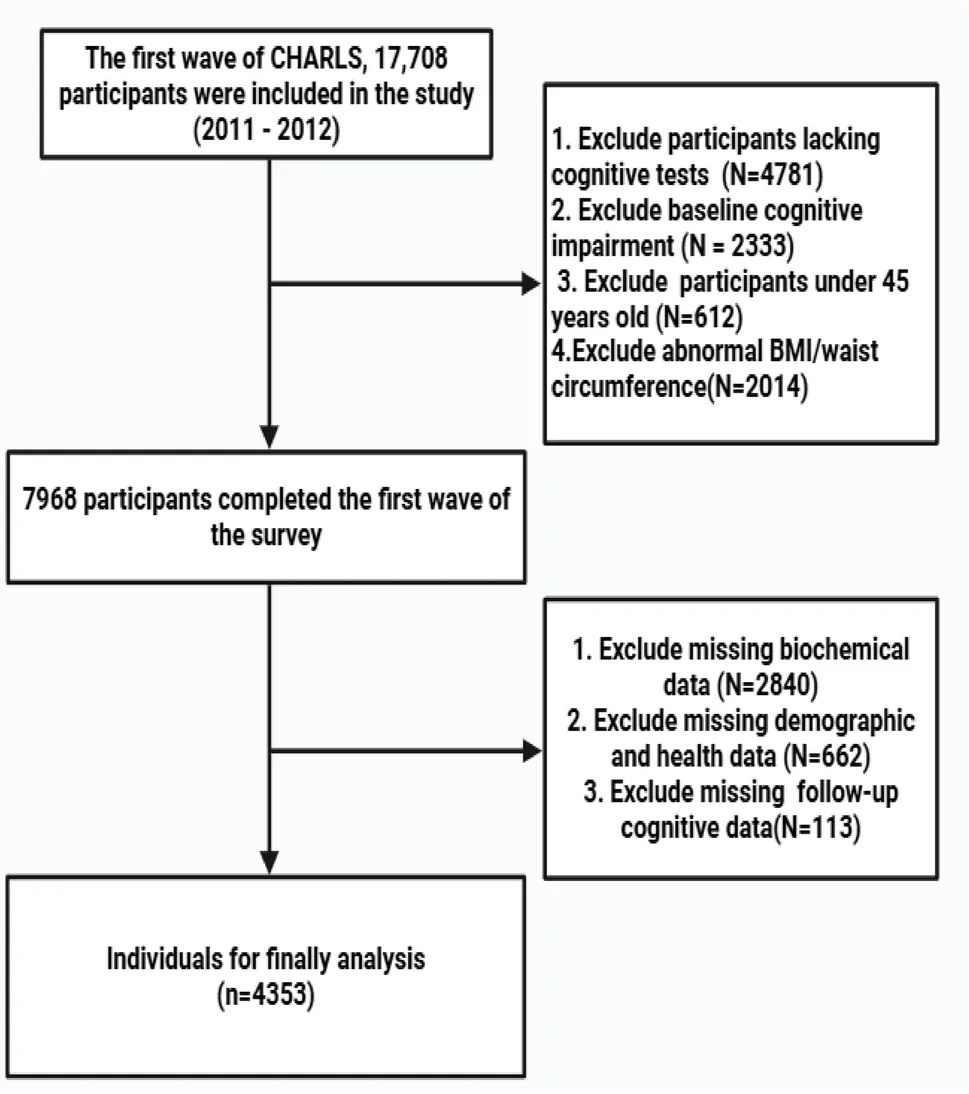
Flowchart of the selection of patients

The analytic cohort included individuals who completed the 2011 baseline evaluation and participated in at least one subsequent cognitive function assessment. Consistent with the STROBE guidelines, the following exclusion criteria were applied: (1) participants younger than 45 years at baseline; (2) those exhibiting cognitive impairment at baseline, thereby restricting the sample to incident cases; (3) individuals with incomplete anthropometric measurements (waist circumference, height, or weight) or biologically implausible outlier values; and (4) those lacking cognitive assessment scores or essential laboratory parameters. Following these exclusions, the final analytic sample comprised 4,353 participants. A schematic flowchart delineating the participant selection and exclusion procedures is presented in Fig. 1.

### Exposure factors and outcome assessment

The main exposure of interest was the modified ABSI, an anthropometric indicator refined to accommodate the specific body composition profiles of Chinese adults. This index includes WC, BMI, and height, offering a more precise measurement of body shape and its health impacts, especially focusing on abdominal fat. Compared with conventional measures, the modified ABSI demonstrates enhanced applicability across heterogeneous groups—including individuals with greater muscularity, Asian populations, and older adults—improving its capacity to predict risks of chronic conditions [13]. Waist circumference was assessed at the end of a normal expiration, whereas height and weight were recorded using standardized calibrated electronic devices. The original ABSI was developed by Krakauer and Krakauer based on the relationship of waist circumference with BMI and height in a U.S. population [14]. Subsequently, Wang et al. developed a modified ABSI formula specifically for Chinese adults based on population-specific body-shape characteristics [15]. The modified ABSI formula used in the present study was calculated as follows:

$$ABSI\left(kg^{-0.731}m^{1.843}\right)=\frac{WC\left(m\right)}{BMI\left(kg/m^{2}\right)^{0.731}\times{height}\:\left(m\right)^{0.619}}$$

The principal endpoint of the present investigation was the occurrence of incident cognitive impairment throughout the follow-up duration. Follow-up commenced at the 2011 baseline examination and extended until the initial documentation of cognitive impairment. Cognitive function was assessed using the cognitive assessment framework applied in CHARLS, which was adapted from the Health and Retirement Study and comprised episodic memory and mental status domains [16, 17]. Two cognitive domains were evaluated: episodic memory and mental status. Episodic memory was assessed using immediate and delayed recall of ten Chinese words. The episodic memory score was calculated as the average number of words correctly recalled in the immediate and delayed recall tasks, ranging from 0 to 10 points [18]. Mental status was assessed using orientation, serial subtraction, and pentagon copying tasks. Orientation was evaluated by asking participants to identify the current year, month, date, day of the week, and season, with one point assigned for each correct response, yielding a score of 0–5 points. Numerical ability was assessed by asking participants to subtract 7 from 100 up to five consecutive times, with one point assigned for each correct subtraction, yielding a score of 0–5 points. Visuospatial ability was assessed in person using a pentagon copying task, in which participants were asked to copy a figure of two overlapping pentagons; a correct copy was assigned 1 point and an incorrect copy was assigned 0 points. Thus, the mental status score ranged from 0 to 11 points. The global cognitive score was calculated as the sum of the episodic memory score and the mental status score, with a total possible range of 0 to 21 points [18]. Following the operational approach used in previous CHARLS-based studies, cognitive impairment was defined as a global cognitive score of < 11 points [12, 19]. Participants with cognitive impairment at baseline were excluded, and incident cognitive impairment was defined as the first follow-up assessment in which the global cognitive score was below 11. This cutoff was used as a screening-based operational criterion for cognitive impairment rather than as a formal clinical diagnosis of dementia.

### Covariates

Baseline covariates were grouped into three categories: demographic and lifestyle characteristics, clinical characteristics, and laboratory parameters. Demographic and lifestyle characteristics included age, sex, residence (city or rural), marital status, educational level, current smoking, and current drinking. Clinical characteristics included self-reported physician diagnoses of hypertension, diabetes mellitus, dyslipidemia, stroke, and heart disease, followed by body mass index (BMI), systolic blood pressure, and diastolic blood pressure. Laboratory measurements were derived from fasting venous blood specimens preserved at − 80 °C and subsequently assayed employing standard enzymatic or immunoturbidimetric techniques. The analysed biomarkers included total cholesterol, triglycerides, HDL-C, LDL-C, HbA1c, high-sensitivity CRP, serum creatinine and uric acid.

### Statistical analysis

Participants were stratified into quartiles according to their modified ABSI values, and the cumulative incidence of cognitive impairment was estimated for each quartile over the 9-year follow-up period. Baseline demographic, clinical, and laboratory characteristics were compared across modified ABSI quartiles. Continuous variables were analyzed using one-way analysis of variance or the Kruskal–Wallis test, as appropriate, whereas categorical variables were analyzed using the chi-square test. To assess potential selection bias related to participant exclusion, baseline characteristics were descriptively compared between participants included in the final analytic cohort and those excluded from the analysis. Standardized mean differences (SMDs) were calculated for continuous and categorical variables to quantify the magnitude of between-group differences. The relationship between modified ABSI and the incidence of cognitive impairment was quantified through Cox proportional hazards regression models, with results expressed as hazard ratios (HRs) and corresponding 95% confidence intervals (CIs). In additional analyses, modified ABSI was also treated as a continuous variable after multiplying the original value by 100 to facilitate interpretation. Its association with incident cognitive impairment was examined using Cox proportional hazards regression models, and the hazard ratio was interpreted as the change in risk associated with each 1-unit increase in modified ABSI × 100. In addition, spearman correlation analyses were performed to evaluate the relationships of continuous BMI, waist circumference, and modified ABSI with cognitive test scores, including episodic memory, mental status, and total cognition. Cognitive score change was calculated as the Wave 3 cognitive score minus the Wave 1 cognitive score. Wave 1 and Wave 3 were selected for this ancillary longitudinal analysis to evaluate cognitive change over a fixed follow-up interval using repeated cognitive assessments available at both time points. Correlation analyses were further performed to examine the relationships of continuous BMI, waist circumference, and modified ABSI with longitudinal changes in episodic memory, mental status, and total cognition.

Three Cox regression models with progressively increasing levels of adjustment were constructed. The crude model was unadjusted. Model 1 was adjusted for age and sex. Model 2 was fully adjusted for age, sex, residence, education, marital status, current smoking, current drinking, hypertension, diabetes mellitus, dyslipidemia, stroke, heart disease, systolic blood pressure, diastolic blood pressure, triglycerides, LDL-C, HDL-C, HbA1c, CRP, creatinine, and uric acid. To examine the potential dose–response relationship between modified ABSI and incident cognitive impairment, restricted cubic spline analyses were performed within Cox proportional hazards regression models. Four knots were placed at prespecified percentiles of the modified ABSI distribution, and the median modified ABSI value was used as the reference. Both unadjusted and fully adjusted models were fitted, and the fully adjusted model included the same covariates as the main Cox regression model. Likelihood ratio tests were used to evaluate the overall association and potential non-linearity, with non-linearity assessed by comparing the spline model with the corresponding linear model. Subgroup analyses were conducted according to prespecified clinically relevant characteristics, including sex, age, hypertension, diabetes mellitus, dyslipidemia, stroke, residence, marital status, current smoking, current drinking, education, and heart disease. Hazard ratios were estimated for each 1-unit increase in modified ABSI ×100, and potential effect modification was assessed using multiplicative interaction terms. In addition, quartile-based sex-stratified Cox regression analyses were performed to further characterize sex-specific associations.

To minimize the potential impact of reverse causation, sensitivity analyses were performed by excluding participants who developed cognitive impairment during the initial 2 years of follow-up. The proportional hazards assumption was assessed using scaled Schoenfeld residuals. Both global and covariate-specific tests were performed at a significance level of 0.05. All statistical operations were executed with R software (version 4.3.2), where a two-tailed *P* value smaller than 0.05 was used to define statistical significance.

## Results

### Clinical characteristics based on ABSI quartiles

Among the 4,353 participants, baseline characteristics differed significantly across ABSI quartiles (Table 1). Higher ABSI scores were associated with older age, as 42.57% of participants in the highest quartile (Q4) were aged ≥ 80 years, and with a greater proportion of females ( *P* < 0.001). The proportion of participants aged ≥ 80 years increased monotonically from Q1 to Q4. Higher ABSI scores were further associated with an adverse cardiometabolic profile, including elevated total cholesterol, low-density lipoprotein cholesterol, glycated hemoglobin (HbA1c), and C-reactive protein (CRP) levels ( *P* ≤ 0.003). A significant inverse trend in BMI was observed across increasing modified ABSI quartiles (*P* < 0.001), with participants in the highest modified ABSI quartile showing the lowest mean BMI. Additionally, the prevalence of hypertension, diabetes, and stroke increased progressively with higher ABSI quartiles ( *P* ≤ 0.032). Participants in the upper ABSI quartiles were more likely to have lower educational attainment, with 37.92% having not completed primary school ( *P* < 0.001). To assess potential selection bias related to participant exclusion, baseline characteristics were compared between participants included in the final analytic cohort and those excluded from the analysis (Supplementary Table 1). Several baseline characteristics differed between the two groups, with the largest standardized mean differences observed for educational attainment (SMD = 0.482), age (SMD = 0.339), marital status (SMD = 0.277), BMI (SMD = 0.213), and sex (SMD = 0.205). These findings indicate that the analytic cohort differed from excluded participants in several observed characteristics and that potential selection bias related to participant exclusion cannot be completely ruled out.

**Table 1:** Baseline Characteristics of Participants Stratified by Quartiles of Modified A Body Shape Index

Variables	Total (*n* = 4353)	Q1 (*n* = 1092)	Q2 (*n* = 1099)	Q3 (*n* = 1086)	Q4 (*n* = 1076)	*P*
Age, *n*(%)						< 0.001
<60	1000 (22.97)	393 (35.99)	281 (25.57)	217 (19.98)	109 (10.13)	
60–69	1114 (25.59)	322 (29.49)	308 (28.03)	267 (24.59)	217 (20.17)	
70–79	1098 (25.22)	226 (20.70)	281 (25.57)	299 (27.53)	292 (27.14)	
≥ 80	1141 (26.21)	151 (13.83)	229 (20.84)	303 (27.90)	458 (42.57)	
Sex, *n*(%)						< 0.001
Female	1933 (44.41)	483 (44.23)	445 (40.49)	440 (40.52)	565 (52.51)	
Male	2420 (55.59)	609 (55.77)	654 (59.51)	646 (59.48)	511 (47.49)	
Residence, *n*(%)						0.126
City	1876 (43.10)	452 (41.39)	455 (41.40)	495 (45.58)	474 (44.05)	
Rural	2477 (56.90)	640 (58.61)	644 (58.60)	591 (54.42)	602 (55.95)	
Marital status, *n*(%)						0.128
Others	529 (12.15)	131 (12.00)	126 (11.46)	120 (11.05)	152 (14.13)	
Married and cohabiting	3824 (87.85)	961 (88.00)	973 (88.54)	966 (88.95)	924 (85.87)	
Education, *n*(%)						< 0.001
Incomplete primary school	1288 (29.59)	264 (24.18)	285 (25.93)	331 (30.48)	408 (37.92)	
Primary school	1164 (26.74)	251 (22.99)	286 (26.02)	306 (28.18)	321 (29.83)	
Middle school	1210 (27.80)	335 (30.68)	339 (30.85)	301 (27.72)	235 (21.84)	
High school and above	691 (15.87)	242 (22.16)	189 (17.20)	148 (13.63)	112 (10.41)	
Current smoking, *n*(%)						0.420
No	2870 (65.93)	711 (65.11)	717 (65.24)	710 (65.38)	732 (68.03)	
Yes	1483 (34.07)	381 (34.89)	382 (34.76)	376 (34.62)	344 (31.97)	
Current drinking, *n*(%)						0.002
No	2716 (62.39)	680 (62.27)	647 (58.87)	670 (61.69)	719 (66.82)	
Yes	1637 (37.61)	412 (37.73)	452 (41.13)	416 (38.31)	357 (33.18)	
Hypertension, *n*(%)						0.032
No	3206 (73.96)	827 (75.73)	816 (74.25)	803 (73.94)	760 (70.63)	
Yes	1129 (26.04)	258 (23.63)	280 (25.48)	277 (25.51)	314 (29.18)	
Diabetes mellitus, *n*(%)						0.001
No	4019 (93.01)	1023 (93.68)	1034 (94.09)	985 (90.70)	977 (90.80)	
Yes	302 (6.99)	62 (5.68)	57 (5.19)	92 (8.47)	91 (8.46)	
dyslipidemia, *n*(%)						0.423
No	3776 (88.10)	953 (87.27)	954 (86.81)	927 (85.36)	942 (87.55)	
Yes	510 (11.90)	119 (10.90)	124 (11.28)	142 (13.08)	125 (11.62)	
Stroke, *n*(%)						0.015
No	4245 (97.79)	1071 (98.08)	1080 (98.27)	1048 (96.50)	1046 (97.21)	
Yes	96 (2.21)	16 (1.47)	17 (1.55)	34 (3.13)	29 (2.70)	
Heart disease, *n* (%)						0.067
No	3818 (87.71)	973 (89.10)	969 (88.17)	956 (88.03)	920 (85.50)	
Yes	535 (12.29)	119 (10.90)	130 (11.83)	130 (11.97)	156 (14.50)	
BMI	23.94 ± 3.69	24.61 ± 4.24	24.31 ± 3.53	23.96 ± 3.42	22.86 ± 3.24	< 0.001
SBP	127.87 ± 20.10	126.70 ± 19.88	126.54 ± 19.44	128.08 ± 19.90	130.21 ± 20.98	< 0.001
DBP	75.58 ± 12.03	76.00 ± 12.25	75.30 ± 12.27	75.68 ± 11.87	75.34 ± 11.72	0.496
Total Cholesterol, Mean ± SD	194.32 ± 38.27	190.09 ± 38.39	194.30 ± 37.04	194.89 ± 38.63	198.07 ± 38.67	< 0.001
Triglycerides, Mean ± SD	135.18 ± 107.78	130.31 ± 115.53	134.63 ± 107.62	134.74 ± 94.91	141.13 ± 111.81	0.136
HDL-C, Mean ± SD	50.22 ± 15.13	51.02 ± 14.94	49.76 ± 14.51	49.85 ± 15.49	50.23 ± 15.54	0.193
LDL-C, Mean ± SD	117.69 ± 35.17	114.41 ± 34.02	118.52 ± 34.44	118.11 ± 35.75	119.74 ± 36.27	0.003
HbA1c, Mean ± SD	5.28 ± 0.82	5.18 ± 0.68	5.27 ± 0.76	5.31 ± 0.87	5.35 ± 0.94	< 0.001
CRP, M (Q₁, Q₃)	1.05 (0.56, 2.13)	0.90 (0.50,1.97)	0.97 (0.54,1.87)	1.05 (0.57,2.14)	1.29 (0.68,2.47)	< 0.001
Creatinine, Mean ± SD	0.80 ± 0.20	0.80 ± 0.17	0.81 ± 0.21	0.81 ± 0.20	0.79 ± 0.20	0.021
Uric acid, Mean ± SD	4.57 ± 1.28	4.53 ± 1.24	4.58 ± 1.24	4.62 ± 1.31	4.56 ± 1.32	0.362

*BMI* body mass index, *SBP* systolic blood pressure, *DBP* diastolic blood pressure, *LDL-C* low-density lipoprotein cholesterol, *HDL-C* high-density lipoprotein cholesterol, *HbA1c* glycated hemoglobin, *CRP* C-reactive protein, *SD* standard deviation

### Association between ABSI and cognitive impairment

The relationship between modified ABSI and the risk of cognitive impairment was quantified using Cox proportional hazards regression models (Table 2). In the crude model, participants in the highest ABSI quartile (Q4) displayed a 28% higher risk relative to those in the lowest quartile (Q1) (HR = 1.28; 95% CI: 1.09–1.51; *P* < 0.001), while the Q3 group also demonstrated a significantly elevated hazard (HR = 1.27; 95% CI: 1.10–1.49; *P* < 0.001). Following adjustment for age and sex in Model 1, ABSI retained its predictive significance. Individuals in Q4 exhibited a 24% increased risk compared with Q1 (HR = 1.24; 95% CI: 1.08–1.42; *P* = 0.002), and those in Q3 showed a 15% elevation (HR = 1.15; 95% CI: 1.01–1.32; *P* = 0.039). In the fully adjusted Model 2, which accounted for a comprehensive set of covariates—including demographic factors, lifestyle behaviors, medical history, and laboratory biomarkers—the independent association of ABSI with cognitive impairment was further confirmed. High ABSI levels were linked to a substantially greater risk independent of conventional metabolic indicators. The most pronounced effect was noted in Q4 (HR = 1.22; 95% CI: 1.04–1.38; *P* = 0.024), with Q3 participants experiencing a 17% higher risk (HR = 1.17; 95% CI: 1.02–1.33; *P* = 0.037); the *P* value for trend was 0.013. No statistically meaningful difference was detected between Q2 and Q1 in any of the models. Kaplan–Meier curves depicted in Supplementary Fig. 1 revealed substantial differences in the cumulative probability of remaining free from cognitive impairment across the four ABSI quartiles over the follow-up period. Individuals assigned to the highest ABSI quartile exhibited the lowest event-free survival probability, in contrast to those in the lowest quartile, who maintained the highest probability of preserving cognitive function. The relative differences between populations were found to be significant by the log-rank test (*P* < 0.001). In additional analyses treating anthropometric indices as continuous variables, modified ABSI remained significantly associated with incident cognitive impairment. Each 1-unit increase in modified ABSI×100 was associated with a higher risk of incident cognitive impairment in the unadjusted model (HR = 1.58, 95% CI: 1.42–1.76, *P* < 0.001), the age- and sex-adjusted model (HR = 1.26, 95% CI: 1.13–1.41, *P* < 0.001), and the fully adjusted model (HR = 1.25, 95% CI: 1.12–1.40, *P* < 0.001) (Supplementary Table 2). Spearman correlation analyses further showed that modified ABSI was inversely correlated with Episodic memory (*r* = − 0.101, *P* < 0.001), Mental status (*r* = − 0.032, *P* < 0.05), and total cognition (*r* = − 0.100, *P* < 0.001), whereas BMI and waist circumference showed weak positive correlations with selected cognitive domains (Supplementary Table 3). We further examined correlations with longitudinal changes in cognitive scores from Wave 1 to Wave 3. Modified ABSI was negatively correlated with changes in Episodic memory (*r* = − 0.04; *P* < 0.05), Mental status (*r* = − 0.08; *P* < 0.001), and total cognition (*r* = − 0.08; *P* < 0.001), whereas BMI showed weak positive correlations with changes in Mental status and total cognition, and waist circumference showed no meaningful correlations (Supplementary Table 4). The Schoenfeld residual tests showed no evidence of violation of the proportional hazards assumption in the Cox regression models. The global test was non-significant (*P* = 0.72), and all covariate-specific tests yielded *P* values greater than 0.05.

**Table 2:** Association between a body shape index and cognitive impairment

Crude model			Model 1		Model 2	
Variables	*P*	HR (95%CI)	*P*	HR (95%CI)	*P*	HR (95%CI)
ABSI						
Q1		1.00 (Reference)		1.00 (Reference)		1.00 (Reference)
Q2	0.137	1.11 (0.97–1.27)	0.506	1.05 (0.91–1.20)	0.428	1.06 (0.92–1.22)
Q3	< 0.001	1.27 (1.10–1.49)	0.039	1.15 (1.01–1.32)	0.037	1.17 (1.00-1.33)
Q4	< 0.001	1.28 (1.09–1.51)	0.002	1.24 (1.08–1.42)	0.024	1.22 (1.04–1.38)
*P* for trend	< 0.001		< 0.001		0.013	

*CI* confidence interval, *HR *hazard ratio

Crude model remained unadjusted

Model 1 incorporated adjustment solely for age and sex

Model 2: adjusted for age, sex, residence, education, marital status, current smoking, current drinking, hypertension, diabetes mellitus, dyslipidemia, stroke, heart disease, systolic blood pressure, diastolic blood pressure, triglycerides, LDL-C, HDL-C, HbA1c, CRP, creatinine, and uric acid

**Fig. 2:**
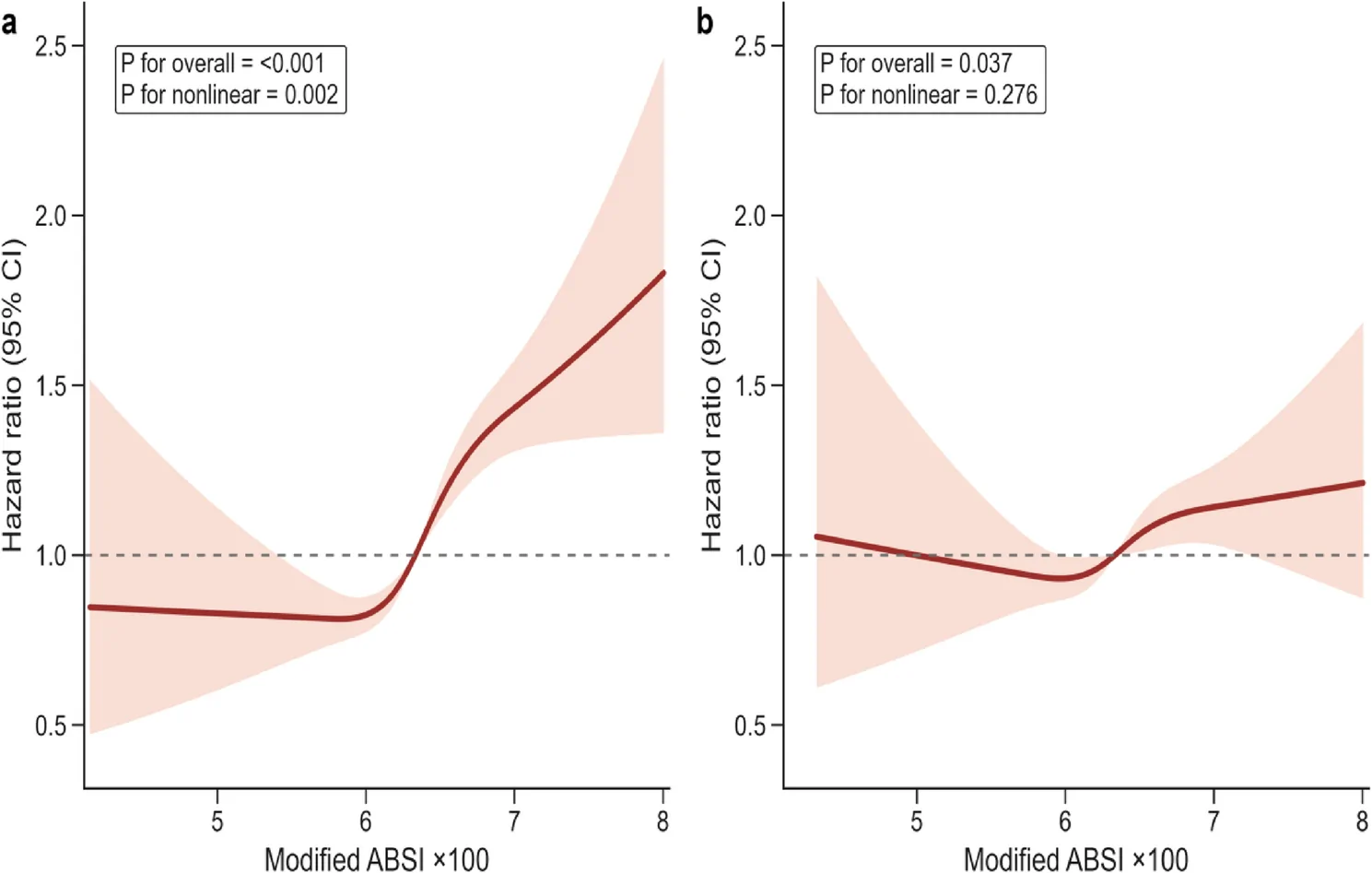
Restricted Cubic Spline Curve. **a** presents the dose–response relationship without adjustment for any covariates, whereas **b** illustrates the association following comprehensive adjustment for age, sex, residence, education, marital status, current smoking, current drinking, hypertension, diabetes mellitus, dyslipidemia, stroke, heart disease, systolic blood pressure, diastolic blood pressure, triglycerides, LDL-C, HDL-C, HbA1c, CRP, creatinine, and uric acid

Restricted cubic spline analyses based on the crude and fully adjusted models are depicted in Fig. 2, revealing the dose–response pattern between modified ABSI and cognitive impairment risk. In the unadjusted analysis, a significant overall association was observed (*P* < 0.001), accompanied by a non-linear component *P*=0.002. Risk remained relatively stable at lower ABSI values but accelerated markedly beyond approximately 6.2, suggesting a potential threshold phenomenon (Fig. 2a). Upon full adjustment for demographic and clinical confounders, the association persisted as statistically significant *P*=0.037, whereas the non-linear term became non-significant *P*=0.276. Consequently, the fully adjusted model supported a linear relationship, whereby the hazard of cognitive impairment increased steadily with rising ABSI levels (Fig. 2b).

### Incidence of cognitive impairment

The analytic sample comprised 4,353 participants at the 2011 baseline, among whom 1,822 incident cases of cognitive impairment were documented by the 2020 follow-up, yielding an overall incidence rate of 41.9% (Supplementary Table 5). Stratification by ABSI quartiles showed 388 cases in Q1, 428 cases in Q2, 469 cases in Q3, and 537 cases in Q4.

### Subgroup analysis

Stratified analyses (Supplementary Fig. 2) demonstrated that elevated modified ABSI levels were linked to a heightened risk of incident cognitive impairment in the entire study population(HR = 1.25; 95% CI: 1.12–1.40; *P* < 0.001). The association remained statistically significant in both women (HR = 1.20; 95% CI: 1.14–1.30) and men (HR = 1.13; 95% CI: 1.02–1.27). Across age strata, statistically significant associations were observed among participants aged 70–79 years and those aged ≥ 80 years, whereas the associations were not statistically significant in the younger age groups. The association was also significant in both urban and rural residents, although the point estimate was numerically higher among urban participants. Similarly, significant associations were observed across most educational strata, except among participants with a high school education or above. To further characterize sex-specific associations, modified ABSI was additionally examined by quartiles within the sex-stratified subgroup analyses. Among female participants, those in the highest modified ABSI quartile had a significantly higher risk of incident cognitive impairment than those in the lowest quartile (Q4 vs. Q1: HR = 1.23; 95% CI: 1.02–1.48; *P* = 0.030) (Supplementary Table 6).

### Sensitivity analysis

Sensitivity analyses were conducted to evaluate the robustness of the primary findings. After exclusion of 874 participants who developed cognitive impairment within the initial two years of follow-up, the association between modified ABSI and incident cognitive impairment persisted as statistically significant (Q4 versus Q1: HR = 1.23, 95% CI: 1.03–1.47; *P* = 0.026) (Supplementary Table 7).

## Discussion

In this prospective cohort study of middle-aged and older Chinese adults, higher modified ABSI was independently associated with an increased risk of incident cognitive impairment. The association remained evident after comprehensive adjustment for demographic, lifestyle, cardiometabolic, cardiovascular, and renal factors and was supported by dose–response, subgroup, and sensitivity analyses. These findings extend previous cross-sectional evidence by demonstrating a prospective association between central body shape and subsequent cognitive impairment.

The present observations are consistent with prior cross-sectional investigations documenting an inverse relationship between ABSI and cognitive performance. For example, the data from the NHANES cohort show high ABSI as cause for low memory and slow processing [20]. Likewise, a cross-sectional evaluation utilizing baseline CHARLS data revealed that higher modified ABSI independently correlated with cognitive impairment [21]. In addition, Mendelian randomization analyses have furnished causal support for the association between abdominal adiposity and cognitive outcomes, demonstrating that each one-standard-deviation increment in BMI-adjusted waist-to-hip ratio (WHRadjBMI) corresponds to a 0.07-standard-deviation reduction in cognitive performance among European populations [22]. By delineating a clear temporal sequence through longitudinal follow-up, the current study extends these earlier findings, confirming that increased ABSI precedes the emergence of cognitive decline. Furthermore, individuals exhibiting higher ABSI levels were more frequently characterized by lower educational attainment. Extant literature suggests that reduced educational attainment may reflect diminished cognitive reserve, thereby constraining the capacity to offset neurodegenerative or metabolic insults, including those associated with central obesity, and consequently heightening susceptibility to cognitive impairment [23–25]. It is noteworthy that certain investigations, such as those conducted in an Iranian cohort, have reported a seemingly “protective” influence of elevated BMI and waist circumference against cognitive deterioration—an observation commonly termed the “obesity paradox.” Such inconsistencies likely arise from the inherent limitations of these traditional metrics in distinguishing visceral adipose tissue from lean muscle mass [26, 27]. The inverse trend observed between BMI and modified ABSI in the present study should be interpreted in light of the construction of modified ABSI. Because modified ABSI normalizes waist circumference by BMI and height, higher modified ABSI does not necessarily indicate greater overall body mass; rather, it reflects a relatively larger waist circumference for a given body size. This finding suggests that modified ABSI may capture body-shape and central-adiposity-related information that is not fully represented by BMI alone [28]. Several biological mechanisms are likely to underlie the observed relationship between modified ABSI and incident cognitive impairment. Visceral adipose tissue operates as an endocrine organ with high metabolic activity, releasing pro-inflammatory mediators such as tumor necrosis factor-α $\:TNF-\alpha\:$ and interleukin-6 (IL-6). These cytokines contribute to persistent low-grade systemic inflammation, which in turn promotes neuroinflammatory processes, neuronal apoptosis, and synaptic disruption within memory-related brain structures, including the hippocampus. The present results lend support to this pathway, as individuals in the highest ABSI quartile displayed markedly higher circulating levels of high-sensitivity C-reactive protein (hsCRP) [29, 30]. Prior investigations have further indicated that central adiposity induces cerebrovascular impairment via oxidative damage to the endothelium and subsequent vascular remodeling, ultimately compromising cerebral perfusion and accelerating cognitive deterioration [8, 31, 32]. Another plausible pathway linking modified ABSI to cognitive impairment may involve blood pressure load, arterial aging, and cardiovascular remodeling. ABSI has been reported to be associated with arterial stiffness, suggesting that this body-shape index may capture vascular aging-related risk beyond general adiposity. In older adults, hypertension and cognitive decline are closely interconnected, and cognitive dysfunction has been recognized as a form of hypertension-mediated target-organ damage [33]. Moreover, arterial aging is characterized not only by increased vascular stiffness but also by structural remodeling of large arteries. Recent evidence indicates that adverse carotid remodeling phenotypes are associated with accelerated age-related central arterial stiffening [34]. Cardiovascular remodeling may also contribute to cognitive vulnerability, as increased left ventricular mass has been associated with cognitive decline and dementia in older adults independently of blood pressure levels [35]. Taken together, these findings suggest that individuals with higher modified ABSI may be more likely to exhibit adverse vascular and cardiac remodeling phenotypes, which may promote cerebral microvascular damage, impaired cerebral perfusion, and ultimately cognitive decline. However, because direct measures of arterial stiffness, carotid remodeling, and left ventricular structure were not available in the present analysis, this mechanistic pathway should be regarded as hypothesis-generating and requires further investigation. Although the interaction by sex was not statistically significant, the numerically higher effect estimate observed in women may be partly related to sex-specific differences in hormonal status and adipose tissue distribution. In middle-aged and older women, the menopausal transition and declining estrogen levels may promote a shift from peripheral subcutaneous fat toward central and visceral fat accumulation [36]. Visceral adiposity is metabolically active and may contribute to insulin resistance, chronic low-grade inflammation, endothelial dysfunction, and cerebrovascular injury, all of which have been implicated in cognitive decline. Therefore, sex-related differences in hormonal milieu and central fat distribution may partly explain the observed numerical variation in the association between modified ABSI and cognitive impairment [37].

Throughout the follow-up interval, the cumulative incidence of cognitive impairment reached 41.9%, highlighting the pressing requirement for straightforward, scalable instruments capable of facilitating early risk identification. Modified ABSI can be computed efficiently from routine anthropometric parameters, rendering it an economical and accessible marker for pinpointing vulnerable middle-aged and older individuals. This attribute is particularly advantageous in primary care environments, where resource-intensive imaging or detailed body composition analyses are often unavailable. Moreover, the findings illuminate the shortcomings of sole reliance on BMI, which may overlook individuals presenting with normal overall body weight yet harboring excessive visceral fat and consequently elevated cognitive risk [38, 39]. Over the longer term, preventive frameworks for healthy cognitive aging should accord greater priority to the management of central obesity and its attendant metabolic derangements. Dietary factors may also contribute to cognitive aging and should be considered. Recent NHANES-based studies have reported associations between vitamin B1 and B2 intake and cognitive performance in older adults. Ji et al. found that higher vitamin B1 intake was associated with lower odds of low cognitive performance across several cognitive domains, while another study reported that higher vitamin B2 intake was associated with better cognitive performance and a lower risk of low cognitive performance [40, 41]. More recently, Wu et al. identified a dose-dependent interaction between dietary vitamins B2 and E, suggesting that nutritional factors may influence cognition not only independently but also through complex nutrient interactions [42]. These findings highlight diet as an important modifiable factor in cognitive health and suggest that unmeasured dietary intake may contribute to residual confounding in observational studies of adiposity and cognitive outcomes.

In summary, the current study establishes modified ABSI as a reliable and independent predictor of cognitive impairment within the aging Chinese population. These observations advocate for the integration of central obesity mitigation into broader public health initiatives targeting dementia prevention. Routine incorporation of ABSI in geriatric evaluations may furnish a more precise and cost-effective approach to risk stratification relative to traditional BMI assessment. Subsequent investigations should emphasize longitudinal interventional trials to ascertain whether targeted reductions in ABSI can successfully postpone or avert cognitive decline. Such endeavors would yield valuable evidence concerning the feasibility of body shape optimization as a practical modality for safeguarding cognitive function in aging cohorts. It capitalizes on the extensive, nationally representative prospective cohort of the CHARLS, which employs multistage stratified sampling to encompass a population broadly reflective of middle-aged and older adults across China. Standardized data collection protocols were implemented, with field investigators receiving specialized training and rigorous quality assurance measures applied to ensure reliable evaluation of exposure variables, potential confounders, and outcome measures. The prospective design further facilitates accurate ascertainment of incident cognitive impairment through extended longitudinal follow-up. Moreover, the present investigation employed suite of statistical techniques to thoroughly delineate the relationship between the modified ABSI and incident cognitive impairment. These approaches encompassed multivariable Cox regression modeling, restricted cubic spline analyses, survival analysis, subgroup analyses, and sensitivity analyses. Nevertheless, several limitations should be acknowledged. First, although we adjusted for a broad range of demographic characteristics, lifestyle factors, chronic diseases, and laboratory biomarkers, residual confounding cannot be completely excluded because of the observational design. Several potentially relevant factors, including dietary patterns, micronutrient intake, medication use, sleep characteristics, subclinical neurological conditions, and other biochemical markers, were unavailable or incompletely captured. In particular, serum phosphate may represent a potential renal-metabolic confounder; however, this variable was unavailable in the CHARLS blood biomarker dataset used in the present analysis, preventing further adjustment. Selection bias due to exclusion of participants with missing baseline measurements or unavailable follow-up data cannot be completely excluded, although we compared baseline characteristics between included and excluded participants. Second, modified ABSI was assessed only at baseline, preventing evaluation of longitudinal changes in body shape, weight trajectories, and their potential associations with cognitive decline. This limitation is particularly relevant in older adults, given age-related alterations in body composition and the potential presence of obesity paradox phenomena. Furthermore, preclinical cognitive decline may contribute to weight loss, reduced appetite, sarcopenia, or frailty before cognitive impairment becomes clinically apparent. Although sensitivity analyses excluding participants who developed cognitive impairment within the first two years of follow-up were performed to reduce potential reverse causation, residual bias related to unmeasured obesity trajectories cannot be fully excluded. Third, cognitive impairment was defined using a composite cognitive score derived from the CHARLS cognitive battery rather than formal clinical diagnostic criteria. Although the cutoff of < 11 has been widely used in previous CHARLS-based studies, it represents an operational screening definition rather than a confirmed diagnosis of dementia. Because clinically adjudicated dementia diagnoses were unavailable, some degree of outcome misclassification may have occurred. Finally, the CHARLS cohort mainly consists of community-dwelling Chinese adults; therefore, caution is warranted when generalizing these findings to institutionalized populations, other ethnic groups, or populations with substantially different body composition patterns and healthcare characteristics. Future studies incorporating repeated anthropometric measurements, comprehensive metabolic assessments, and clinically adjudicated cognitive outcomes are needed to further validate these findings.

## Conclusion

The present prospective analysis, drawing upon data from the CHARLS cohort, substantiates that elevated modified ABSI constitutes an independent risk factor for incident cognitive impairment in middle-aged and older adults. The observed association between increasing modified ABSI and cognitive impairment risk suggests that central body-shape characteristics may provide information relevant to cognitive aging beyond that captured by traditional weight-based measures. Future studies should endeavor to elucidate the biological pathways mediating this association, examine whether serial ABSI measurements can refine risk prediction, and evaluate the long-term efficacy of interventions directed at central adiposity reduction in preserving cognitive function. Public health programs to address the increasing problem of cognitive impairment for aging populations would benefit from incorporating ABSI in everyday community-based health screenings. Such incorporation could complement existing measures like BMI and waist circumference, thereby enabling earlier identification and timely preventive interventions for at-risk individuals.

## Supplementary Information

Supplementary Material 1.

## Data Availability

The datasets analysed during the current study are available from the China Health and Retirement Longitudinal Study (CHARLS) repository at https://charls.pku.edu.cn/. Access to the datasets is available after user registration and approval by the CHARLS research team.

## Abbreviations

ABSI: A Body Shape Index
BMI: body mass index
CHARLS: China Health and Retirement Longitudinal Study
CI: confidence interval
CRP: C-reactive protein
DBP: diastolic blood pressure
HbA1c: glycated haemoglobin
HDL-C: high-density lipoprotein cholesterol
HR: hazard ratio
KM: Kaplan–Meier
LDL-C: low-density lipoprotein cholesterol
RCS: restricted cubic spline
SBP: systolic blood pressure
SD: standard deviation
SMD: standardized mean difference
WC: waist circumference
Q1–Q4: first to fourth quartiles
