# Supplementary material for: Association between modified a body shape index and incident cognitive impairment in middle-aged and older adults: a prospective cohort study
Source: BMC Public Health. 2026 Aug 17;26:3352. doi: 10.1186/s12889-026-28763-6 (PMC13756848; doi:10.1186/s12889-026-28763-6)
Supplement: Supplementary file 1 [file 12889_2026_28763_MOESM1_ESM.docx]

**Association between Modified A Body Shape Index and Incident Cognitive Impairment in Middle-aged and Older Adults: A Prospective Cohort Study**

Supplementary Table 1. Comparison of baseline characteristics between included and excluded participants

| Variable | Included participants (n = 4,353) | Excluded participants (n = 13,355) | SMD |
| --- | --- | --- | --- |
| Age, n (%) |  |  | 0.339 |
| <60 | 1000 (22.97) | 4670 (34.97) |  |
| 60–69 | 1114 (25.59) | 3747 (28.06) |  |
| 70–79 | 1098 (25.22) | 2930 (21.94) |  |
| ≥80 | 1141 (26.21) | 2008 (15.03) |  |
| Sex, n (%) |  |  | 0.205 |
| Female | 1933 (44.41) | 7294 (54.62) |  |
| Male | 2420 (55.59) | 6059 (45.38) |  |
| Residence, n (%) |  |  | 0.070 |
| City | 1876 (43.10) | 5295 (39.65) |  |
| Rural | 2477 (56.90) | 8060 (60.35) |  |
| Marital status, n (%) |  |  | 0.277 |
| Others | 529 (12.15) | 3009 (22.53) |  |
| Married and cohabiting | 3824 (87.85) | 10346 (77.47) |  |
| Education, n (%) |  |  | 0.482 |
| Incomplete primary school | 1288 (29.59) | 7097 (53.20) |  |
| Primary school | 1164 (26.74) | 2505 (18.78) |  |
| Middle school | 1210 (27.80) | 2261 (16.95) |  |
| High school and above | 691 (15.87) | 1477 (11.07) |  |
| Current drinking, n (%) |  |  | 0.133 |
| No | 2716 (62.39) | 9069 (68.71) |  |
| Yes | 1637 (37.61) | 4130 (31.29) |  |
| Current smoking, n (%) |  |  | 0.154 |
| No | 2870 (65.93) | 9166 (73.01) |  |
| Yes | 1483 (34.07) | 3388 (26.99) |  |
| Hypertension, n (%) |  |  | 0.003 |
| No | 3206 (73.96) | 9729 (74.07) |  |
| Yes | 1129 (26.04) | 3406 (25.93) |  |
| Diabetes mellitus, n (%) |  |  | 0.048 |
| No | 4019 (93.01) | 12327 (94.19) |  |
| Yes | 302 (6.99) | 760 (5.81) |  |
| Dyslipidemia, n (%) |  |  | 0.089 |
| No | 3776 (88.10) | 11741 (90.83) |  |
| Yes | 510 (11.90) | 1186 (9.17) |  |
| Stroke, n (%) |  |  | 0.047 |
| No | 4245 (97.79) | 12795 (97.04) |  |
| Yes | 96 (2.21) | 390 (2.96) |  |
| Heart disease, n (%) |  |  | 0.005 |
| No | 3818 (87.71) | 11542 (87.86) |  |
| Yes | 535 (12.29) | 1595 (12.14) |  |
| BMI, Mean ± SD | 23.94 ± 3.69 | 23.18 ± 3.42 | 0.213 |
| Systolic blood pressure, Mean ± SD | 127.87 ± 20.10 | 129.45 ± 18.98 | 0.081 |
| Diastolic blood pressure, Mean ± SD | 75.58 ± 12.03 | 75.28 ± 10.39 | 0.027 |
| Total cholesterol, Mean ± SD | 194.32 ± 38.27 | 191.02 ± 29.06 | 0.097 |
| Triglycerides, Mean ± SD | 135.18 ± 107.78 | 119.98 ± 68.84 | 0.168 |
| HDL-C, Mean ± SD | 50.22 ± 15.13 | 50.20 ± 11.44 | 0.001 |
| LDL-C, Mean ± SD | 117.69 ± 35.17 | 114.47 ± 25.72 | 0.105 |
| HbA1c, Mean ± SD | 5.28 ± 0.82 | 5.20 ± 0.62 | 0.111 |
| CRP, Median (Q1, Q3) | 1.05 (0.56, 2.13) | 1.00 (0.88, 1.30) | 0.077 |
| Creatinine, Mean ± SD | 0.80 ± 0.20 | 0.77 ± 0.20 | 0.130 |
| Uric acid, Mean ± SD | 4.57 ± 1.28 | 4.39 ± 0.98 | 0.161 |

**Supplementary Table 2: Modified A Body Shape Index as a Continuous Variable**

| Model | HR (95% CI) | *P* |
| --- | --- | --- |
| Crude Model | 1.58 (1.42–1.76) | <0.001 |
| Model 1 | 1.26 (1.13–1.41) | <0.001 |
| Model 2 | 1.25 (1.12–1.40) | <0.001 |

Note: The crude model was unadjusted. Model 1 was adjusted for age and sex. Model 2 was adjusted for age, sex, residence, education, marital status, current smoking, current drinking, hypertension, diabetes mellitus, dyslipidemia, stroke, heart disease, systolic blood pressure, diastolic blood pressure, triglycerides, LDL-C, HDL-C, HbA1c, CRP, creatinine, and uric acid. CI, confidence interval; HR, hazard ratio.

**Supplementary Table 3. Correlations of Baseline BMI, Waist Circumference, and Modified ABSI with Cognitive Scores**

| Variable | Episodic memory | Mental status | Total cognition |
| --- | --- | --- | --- |
| BMI | 0.084*** | 0.040* | 0.092*** |
| Waist circumference | 0.023 | 0.072*** | 0.067*** |
| Modified ABSI | −0.101*** | −0.032* | −0.100*** |

Note: Correlations were assessed using Spearman rank correlation coefficients.*P < 0.05, **P < 0.01, ***P < 0.001.

### **Supplementary Table 4. Correlations of Baseline BMI, Waist Circumference, and Modified ABSI with Changes in Cognitive Scores from Wave 1 to Wave 3**

| Variable | Episodic memory change | Mental status change | Total cognition change |
| --- | --- | --- | --- |
| BMI | 0.02 | 0.05** | 0.05** |
| Waist circumference | 0.01 | 0.01 | 0.01 |
| Modified ABSI | −0.04* | −0.08*** | −0.08*** |

**Note:** Cognitive score change was calculated as the Wave 3 score minus the Wave 1 score. Correlations were assessed using Spearman rank correlation coefficients.*P < 0.05, **P < 0.01, ***P < 0.001.

**Supplementary Table 5. Incidence of cognitive impairment during follow-up by ABSI group**

| ABSI | Numbers | Events | Incidence rate |
| --- | --- | --- | --- |
| Total | 4353 | 1822 | 41.90% |
| Q1 | 1092 | 388 | 35.50% |
| Q2 | 1099 | 428 | 38.90% |
| Q3 | 1086 | 469 | 43.20% |
| Q4 | 1076 | 537 | 49.90% |

**Supplementary Table 6.Association between modified A Body Shape Index and cognitive impairment by sex**

| Female | | | Male | |
| --- | --- | --- | --- | --- |
| Variables | *P* | HR (95%CI) | *P* | HR (95%CI) |
| ABSI |  |  |  |  |
| Q1 |  | 1.00 (Reference) |  | 1.00 (Reference) |
| Q2 | 0.217 | 1.19 (0.90-1.56) | 0.770 | 1.03 (0.82-1.30) |
| Q3 | 0.064 | 1.20 (0.99 ~ 1.45) | 0.192 | 1.16(0.93-1.46) |
| Q4 | 0.030 | 1.23 (1.02 ~ 1.48) | 0.135 | 1.20 (0.95-1.52) |

Note：Adjusted for age, residence, education, marital status, current smoking, current drinking, hypertension, diabetes mellitus, dyslipidemia, stroke, heart disease, systolic blood pressure, diastolic blood pressure, triglycerides, LDL-C, HDL-C, HbA1c, CRP, creatinine, and uric acid.

**Supplementary Table 7. Association of Modified A Body Shape Index with Incident Cognitive Impairment in Sensitivity Analysis(N=3479)**

| Variables | *P* | HR (95%CI) |
| --- | --- | --- |
| ABSI |  |  |
| Q1 |  | 1.00 (Reference) |
| Q2 | 0.294 | 1.10 (0.92-1.31) |
| Q3 | 0.117 | 1.15 (0.97-1.38) |
| Q4 | 0.026 | 1.23 (1.03-1.47) |

Note：Adjusted for age, sex, residence, education, marital status, current smoking, current drinking, hypertension, diabetes mellitus, dyslipidemia, stroke, heart disease, systolic blood pressure, diastolic blood pressure, triglycerides, LDL-C, HDL-C, HbA1c, CRP, creatinine, and uric acid.

**Supplementary Figure 1. Cumulative Hazard Curves by Modified A Body Shape Index (ABSI) Quartiles**

**Supplementary Figure 2. Forest Plot Depicting the Association Between Modified A Body Shape Index (ABSI) and Cognitive Impairment Across Subgroup Analyses.**
